# Illness perceptions of adults with eczematous skin diseases: a systematic mixed studies review

**DOI:** 10.1186/s13643-021-01687-5

**Published:** 2021-05-07

**Authors:** Marc Rocholl, Michaela Ludewig, Carola Brakemeier, Swen Malte John, Annika Wilke

**Affiliations:** 1 Institute for Health Research and Education, Department of Dermatology, Environmental Medicine and Health Theory, University of Osnabrueck, Am Finkenhuegel 7a, 49076 Osnabrueck, Germany; 2 Institute for Interdisciplinary Dermatological Prevention and Rehabilitation (iDerm), University of Osnabrueck, Am Finkenhuegel 7a, 49076 Osnabrueck, Germany

**Keywords:** Illness perceptions, Self-regulation model, Eczema, Atopic dermatitis, Contact dermatitis, Systematic review

## Abstract

**Background:**

Eczematous skin diseases, e.g., atopic dermatitis or contact dermatitis, are associated with a high disease burden, a significant impact on quality of life and a higher risk for anxiety and depression. Therefore, coping strategies are of interest. In order to understand coping processes, it is necessary to examine the patients’ perspectives on their illness. The aim of this systematic mixed studies review is to investigate the illness perceptions of patients with eczematous skin diseases to get a better understanding of their coping processes.

**Methods:**

We performed a systematic literature search in PubMed, The Cochrane Library, PsycInfo, PSYNDEX, CINAHL, Web of Science, and Scopus until February 20, 2019. Both qualitative and quantitative studies were included in the review. Two independent reviewers conducted data extraction and carried out a narrative synthesis. We assessed study quality with the Mixed Methods Appraisal Tool.

**Results:**

Three qualitative and four quantitative studies were included in the systematic review. We found different methodological approaches for investigating illness perceptions: guided interviews, focus group interviews as well as standardized questionnaires, e.g., the Brief Illness Perception Questionnaire. All studies report suspected causes of the skin disease, such as endogenous and exogenous causes (namely, psychological or occupational factors). We found long timeline beliefs as well as various perceived and experienced social, economic, and psychological consequences. Our analysis reveals complex emotional representations in patients with eczematous skin diseases, in particular impairment of emotional well-being, and feelings of shame or helplessness. Qualitative and quantitative data were predominantly complementary and convergent.

**Conclusion:**

Patients with eczematous skin diseases have complex illness representations regarding their disease. These representations interrelate with the coping behavior of patients. Therefore, medical professionals should consider them for counseling and treatment.

**Systematic review registration:**

PROSPERO 2018 CRD42018109217.

**Supplementary Information:**

The online version contains supplementary material available at 10.1186/s13643-021-01687-5.

## Background

Eczema is a very common skin condition. It predominantly comprises of atopic dermatitis (AD, syn. atopic eczema (AE), eczema) and contact dermatitis (CD), which are henceforth referred to as eczematous skin diseases (ESD). AD is usually characterized by chronic or chronically relapsing skin inflammation with onset frequently already in early childhood and is associated with dry skin and intensive pruritus [1, 2]. It is primarily an endogenous disease, but can be pivotally influenced by environmental factors [3]. However, its etiology is not completely understood yet [4]. CD comprises irritant contact dermatitis (ICD) and allergic contact dermatitis (ACD). It is caused by occupational or non-occupational skin exposure to irritants and/or contact allergens, respectively [5]. Avoidance of the causative agent(s) may lead to clearance of skin symptoms. If detection of the causative agent(s) fails or its complete avoidance is not possible, CD may also result in a chronic condition [6].

For the individual patient, the burden of disease is usually high due to soreness or itching, sleep disorders, feelings of stigmatization, restraints on leisure activities, prolonged sick leave from work or school, impaired social contact, and time consuming treatment, just to name a few [4, 7–11]. Studies indicate a significant and long-term impact on quality of life (QoL) of patients with AD [8, 10, 12], contact dermatitis [7], and (especially work-related) ACD [13–15]. In addition, several studies found higher rates of anxiety and depression among AD patients [10, 16–19].

Against this background, coping with ESD is of particular importance. In order to understand coping processes, defined as the processes of adapting to health threats (e.g., adherence to treatment), it is necessary to examine the patients’ perspectives on their illness [20, 21]. These so-called illness perceptions (or illness representations, e.g., people’s understanding of their illness) are essential, because they have a large impact on health behavior, especially with regard to coping responses and illness management [21, 22]. Leventhal’s C*ommon-Sense Model of Self-Regulation of Health and Illness* (*CSM*) [20, 23] is a valuable framework to describe and understand choosing and planning of coping processes. It assumes individual cognitive and emotional representations, both independently, to be key determinants to evaluate health and illness [22, 24]. Several studies report correlations of illness perceptions with pivotal behavioral and quality-of-life outcomes [25]. In their meta-analytic review, Hagger and Orbell [25] found that perceived serious consequences, high identity beliefs and expected chronic timeline of the illness are associated with impaired psychological well-being, role and social functioning, more psychological distress as well as decreased vitality. Additionally, higher psychological well-being, social functioning, and vitality were associated with higher control beliefs [25]. Broadbent et al. [26] systematically reviewed the usage of the Brief Illness Perception Questionnaire (B-IPQ) [27] in various illnesses and outlined strong associations between depression and anxiety together with lower quality-of-life dimensions, and serious perceived consequences, emotional representations, and strong identity. Higher personal and treatment control beliefs were found to be negatively associated with depression and anxiety and, furthermore, positively associated with better quality of life [26].

The illness representations of various skin diseases (for instance psoriasis, including psoriasis arthritis [28–30], vitiligo [31], and alopecia areata [32]) are thoroughly investigated. However, there is no systematic review on the illness representations of patients with ESD. The aim of this systematic mixed studies review was therefore to describe the representations of health and illness for this patient population.

## Methods

This review was registered in the International Prospective Register of Systematic Reviews on September 18, 2018 (PROSPERO registration number CRD42018109217) [33]. This report follows the PRISMA statement (Preferred Reporting Items for Systematic Reviews and Meta-Analyses) [34]. A completed PRISMA checklist is attached in Additional file 1.

### Information sources and search strategy

We performed a systematic literature search using controlled vocabulary and text words related to eczematous skin diseases and illness perceptions without any language limitations from date of inception until February 20, 2019, in the following databases: MEDLINE (via PubMed), The Cochrane Library, PsycInfo (via EBSCO host), PSYNDEX (via EBSCO host), CINAHL (via EBSCO host), Web of Science Core Collection, and Scopus. Full electronic search strategies are attached in Additional file 2. The systematic literature search in MEDLINE (via PubMed) was updated on 18 March 2020, and led to another 228 results, of which none met the inclusion criteria. We conducted hand search for gray literature (e.g., via Google Scholar and OpenGrey). Reference lists of the studies included were independently screened by two reviewers to identify additional eligible reports (backward citation tracking). In addition, we conducted forward citation tracking via Web of Science Core Collection. If studies were not indexed in Web of Science Core Collection, we used Google Scholar to check citing references. Search strategies were developed by MR and discussed with ML, AW, and CB. The first author performed all database searches.

### Inclusion and exclusion criteria

This systematic review considers qualitative and quantitative studies as well as mixed-method approaches. Only full-text journal articles were included. We excluded editorials, comments, case reports, conference abstracts, letters, and book chapters. Studies reported in German or English language were included in the review. Studies written in other languages are listed in Additional file 3. Studies were suitable for inclusion if study participants were at least 18 years old and had a medically confirmed diagnosis of ESD (AD, ICD, ACD, or mixed diagnoses). Studies focusing on parents of affected children, medical professionals (e.g., physicians, nurses), and studies focusing on patients with other skin disorders (e.g., psoriasis, seborrheic dermatitis, or pruritus) were excluded. The phenomenon of interest of this review is illness perceptions. Hence, we included studies reporting any outcome that can be assigned to at least one of Leventhal’s dimensions of CSM. Studies exclusively focusing on self-assessment of severity, quality of life, or depression were also excluded. In order to facilitate the study selection process, the aforementioned criteria were listed in tabular form (see Additional file 4).

### Study selection

**Fig. 1:**
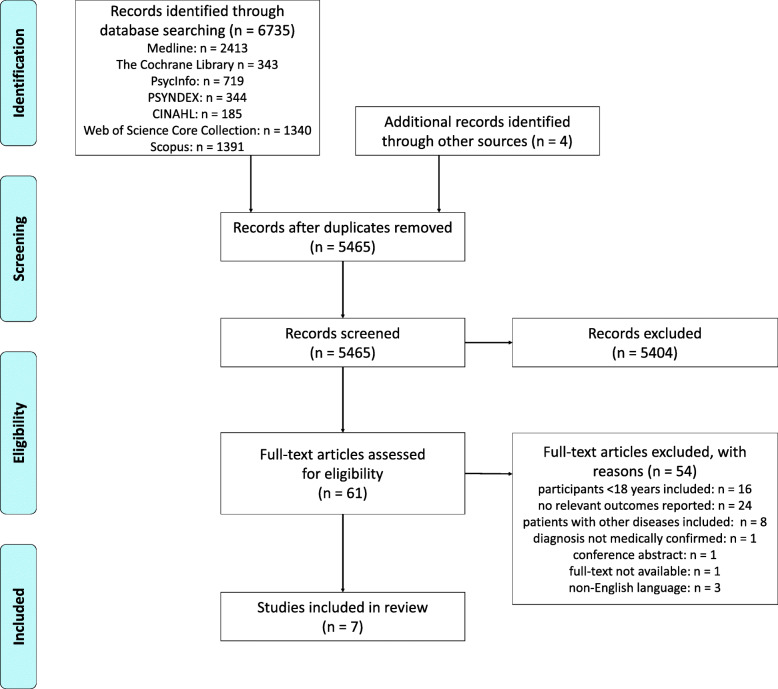
PRISMA flow chart of search and study selection

After removing duplications with EndNote X9, three reviewers (MR/ML and CB) independently examined titles and abstracts for eligibility by using Rayyan [35]. Subsequently, the full texts of potentially eligible studies were reviewed and checked for inclusion suitability by two reviewers (MR and CB) independently. Disagreements were resolved through discussion and consensus. If necessary, we requested additional information from the study authors in order to clarify questions on eligibility. Results of the screening process are reproduced in a PRISMA flow chart (see Fig. 1).

### Data extraction

Two reviewers (MR and CB) independently performed the data extraction process using pre-defined Excel spreadsheets in duplicate. Disagreements were resolved by discussion between the two reviewers. If necessary, a third reviewer was consulted. In case of missing datasets or unclear information, we contacted the corresponding authors and asked for further information. Extracted data include information on (1) study characteristics (study design, funding, setting, inclusion and exclusion criteria, country, and study objectives), (2) study participants (population, sample size, age, sex ratio, diagnoses), (3) methodology (data collection method, underlying theory), and (4) all reported relevant outcomes regarding the five attributes of illness perceptions as described in Leventhal’s CSM [20, 23], assessed by either qualitative (e.g., interviews) or quantitative (e.g., questionnaires) research methods. The data from the qualitative studies were primarily extracted on the theme level. Whenever possible, these were supplemented by extracted verbatim quotations, with the aim of providing more details on the results. In addition to extracting percentages on age and sex distribution from all included studies, primarily means and standard deviations were extracted from the quantitative studies. In order to prevent extraction errors, the extracted data were reviewed by an additional experienced researcher (ML).

### Data synthesis

**Fig. 2:**
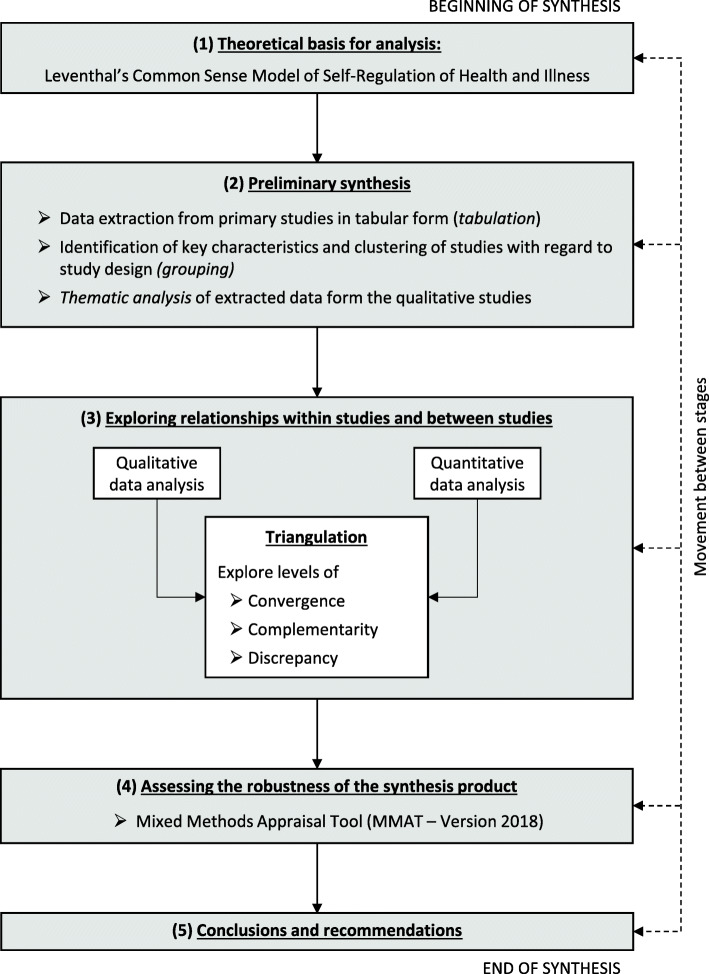
Narrative synthesis process. Modified according to Popay et al. [36] and Arai et al. [37]

We assumed that outcomes, study populations, and study designs would be too heterogeneous to apply statistical methods only and thus a narrative synthesis would be appropriate. Data synthesis was carried out in four steps following the guidance of Popay et al. [36] and Arai et al. [37]. However, the approach was adjusted according to the research questions and the topic of this review (see Fig. 2).

#### Theoretical basis for analysis

As outlined by Arai et al. [37], an underlying theoretical framework facilitates integrating results from the various analysis steps. We used Leventhal’s *Common Sense Model of Self-Regulation of Health and Illness* [20, 23] as underlying theory as it “provides a framework for describing and understanding the processes involved in the initiation and maintenance of behaviors for managing illness threats” (page 936) [24].

**Fig. 3:**
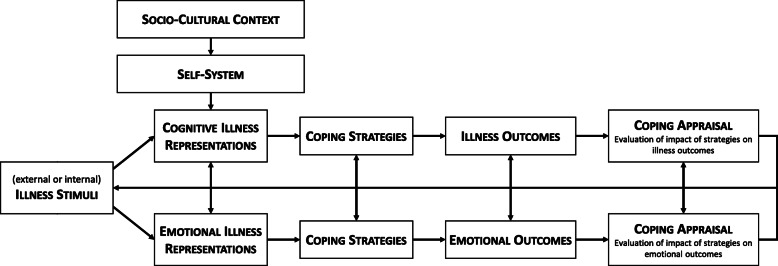
Leventhal’s common sense model of self-regulation (adapted from [20, 25, 38])

The basic assumption of the framework is that potential or actual health threats experienced by persons (e.g., symptoms) lead to a parallel processing self-regulation process on the cognitive and emotional level (see Fig. 3). People develop own perspectives in order to understand illness threats. These cognitive illness representations are manifested in five contiguous dimensions: *Identity* (the label and the symptoms assigned to the disease); *Cause* (individual’s beliefs of the cause of the illness); *Timeline* (the expected time of development, duration and recovery—e.g., acute, chronic, or cyclical); *Consequences* (expected and perceived consequences in terms of several dimensions—e.g., social, economic, physiological, and psychological); *Controllability* (assumptions about the amount of personal control and treatment control over a disease). Over time, another dimension called *Illness Coherence* (the extent to which the disease is comprehensible to someone) has been integrated in the CSM. These dimensions are substantially responsible for choosing and planning coping behavior. In the CSM, it is assumed that individuals evaluate the success of chosen coping behavior and, if necessary, modify their own illness perceptions. Illness representations can be measured with various instruments, of which the Illness Perception Questionnaire (IPQ) [39] and its revised version (Revised Illness Perception Questionnaire; IPQ-R) [40] are commonly used. In addition, a short version of the questionnaire—the Brief Illness Perception Questionnaire (B-IPQ) [27]—is available.

#### Preliminary synthesis

We used tabulation of data to identify key aspects of the studies and thus become familiar with them. Taking into consideration that tabulation allows the identification of study characteristics, which facilitate the organization of the studies in different groups and simplify subsequent analysis steps, we clustered the studies with regard to the study design. As mentioned by Arai et al. [37] and Popay et al. [36], thematic analysis is a common technique to analyze data from qualitative studies in systematic reviews. We therefore used thematic analysis with both, theoretically driven and inductive approaches, to systematically identify major themes across qualitative studies.

#### Exploring relationship within studies and between studies

After analyzing qualitative and quantitative data separately, the results were examined for convergence, complementarity, and discrepancies. To explore the relationships within and across data drawn from the studies, we applied *methodological triangulation* [41] according to Forster [42] and O’Cathain [43], who define triangulation as a research process, which combines different research approaches in order to generate a more comprehensive, multidimensional perspective on a phenomenon. It should be noted, however, that the concept of triangulation is not consistently defined in literature.

#### Assessing robustness of the synthesis

Different study designs usually require the use of different quality assessment tools. We chose the Mixed Methods Appraisal Tool (MMAT) [44, 45] for validity assessment in our review as it is a very useful tool to estimate the methodological quality of quantitative, qualitative, and mixed-method studies. It thus can be applied for assessing quality of all included studies. Two authors (MR and ML) independently assessed methodological quality of each study and afterwards reflected critically on the synthesis process.

## Results

### Results of the search

A total of 6735 records were initially identified through database searches. In addition, four records have been added from other sources. After removal of duplications, 5465 records were screened (titles and abstracts) down to 61 potentially eligible studies. Reviewing full texts led to exclusion of another 54 records. Of the 5465 records initially screened, seven met the inclusion criteria. Figure 1 presents the PRISMA flow diagram with the number of included and excluded studies.

### Description of studies

We included three studies with qualitative research approach (two studies using semi-structured guided interviews [46, 47], one study reporting results of semi-structured focus group interviews [48]). Four studies assessed illness perception with quantitative methods, all with cross-sectional design [49–52]. The studies included were conducted in Canada [47], Czech Republic [52], Denmark [48, 49], Germany [46], Israel [51], and the United Kingdom (UK) [50]. The study characteristics are summarized in Table 1.

**Table 1:** Study characteristics of included studies

Qualitative research approach						
	Sample size	Sex % (male/female)	Age (mean, SD; range)	Diagnosis	Research methods	Setting
Bathe et al. [46] *Germany*	50	60.0/40.0	44.4, SD=11.5; range: N/A	Occupational skin disease (not specified)	Semi-structured guided interviews	Occupational rehabilitation clinic
Mollerup et al. [48] *Denmark*	23	48.0/52.0	45.8, SD=14.2; range: N/A	AD; AHE; IHE	Semi-structured focus group interviews	Tertiary referral center
Zack et al. [47] *Canada*	14	57.1/42.9	45.0, SD=N/A; range: 20-64	(Work-related and non-work related) CD (incl. ACD, ICD; A&ICD; I&ATCD; ECD);	Semi-structured guided interviews	Occupational health clinic
Benyamini et al. [51] *Israel*	303^a^	36.6/63.4	46.0, SD=16.0; range: N/A	CD (incl. ACD and ICD); AD (acc. to Simpson and Hanifin [53]); OD (acc. to Mathias’s criteria [54])	B-IPQ	Tertiary referral center
Březinová et al. [52] *Czech Republic*	128	40.6/59.4	30.2, SD=9.8; range: 18-61	AD (acc. to Hanifin-Rajka’s criteria [55])	B-IPQ	University hospital
Mollerup et al. [49] *Denmark*	294	35.4/64.6	N/A, SD=N/A; range: 18-69	AD; AHE; IHE (information extracted from [56])^b^	(modified) NOSQ-2002 [57]	Tertiary referral center; secondary referral center
Wittkowski et al. [50] *UK*	284	24.7/75.3	23, SD=N/A; range: 18-66	AD	IPQ-R	General population (members of the National Eczema Society (NES); students of the University of Manchester)

*ACD* allergic contact dermatitis, *AD* atopic dermatitis, *A&ICD* allergic and irritant contact dermatitis, *AHE* allergic hand eczema, *B-IPQ* Brief Illness Perception Questionnaire, *CD* contact dermatitis, *ECD* endogenous contact dermatitis, *HE* hand eczema, *ICD* irritant/irritative contact dermatitis, *IHE* irritant/irritative hand eczema, *I&ATCD* irritant/irritative and atopic contact dermatitis, *NES* National Eczema Society, *NOSQ-2002* Nordic Occupational Skin Questionnaire, *OD* occupational dermatitis, *IPQ-R* revised Illness Perception Questionnaire

^a^Benyamini et al. [51] report further data on a fourth (control) group (*n* = 82), which covers other diseases, e.g., asteatotic eczema, vesicular, hand eczema, granuloma annulare, seborrheic dermatitis, rosacea, lichen sclerosus et atrophicus, and psoriasis. The results reported in this review refer exclusively to the three groups listed in Table 1. All values of the control group were excluded from the following analyses and thus, a total sample size of *n* = 221 is stated in Table 2

^b^The diagnoses of the study participants are not described in detail in the publication of Mollerup et al. [49]. After correspondence with the first author of the paper, diagnoses could be derived from another source [56]

Participant numbers in the qualitative studies ranged from 14 [47] to 50 [46] with a broadly similar sex distribution and similar age (mean age across studies around 44.8 years). A total of 926 participants were investigated in the quantitative studies. Regarding age and sex distribution, the results were more heterogeneous. The percentage of male participants varied between 24.7% [50] and 40.6% [52]. In addition, the average age (means range from 23.0 years [50] of 46.0 years [51]) is lower compared to the qualitative data. One study fails to report the average age of the participants [49]. Except one, all quantitative data were collected in a clinical setting. Only Wittkowski et al. [50] collected data from members of the National Eczema Society (NES, UK) and students from the University of Manchester.

While Bathe et al. [46] reported exclusively patients with work-related skin diseases, Mollerup et al. [48] and Zack et al. [47] investigated both, work-related and non-work related ESD. Overall, diagnoses within the included studies vary. Two studies exclusively focus on patients with AD [50, 52], whereas four studies summarized different diagnoses in their samples [47–49, 51]. Only one study [46] did not report details on participants’ diagnoses.

### Identity

**Fig. 4:**
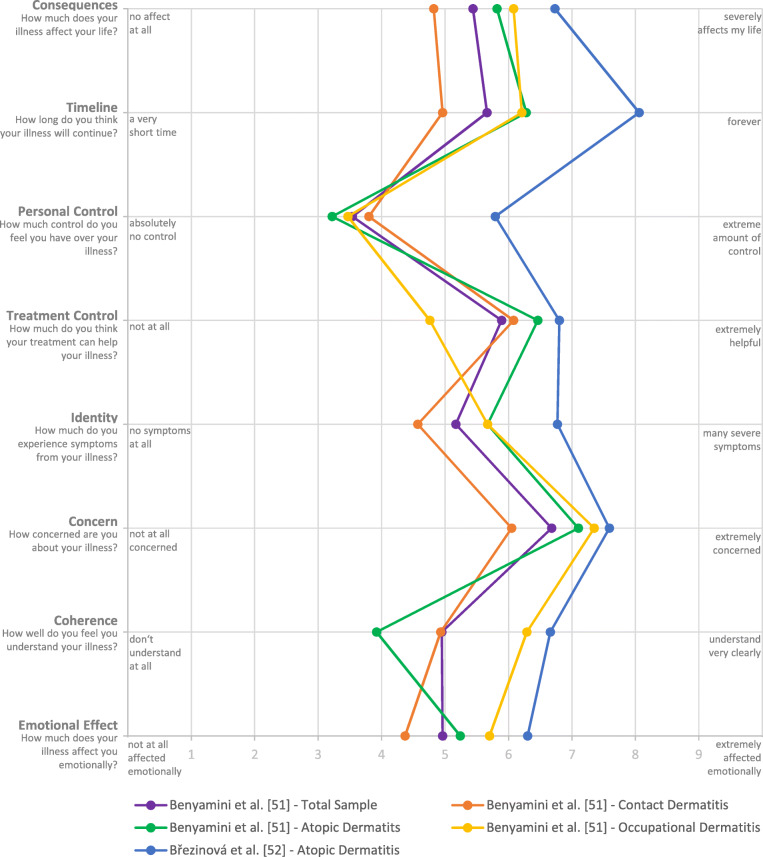
Mean values of the Brief Illness Perception Questionnaire [51, 52]

The identity dimension of a disease contains the label or name as well as symptoms that are characteristically for the disease. The following symptoms could be extracted from included qualitative studies: redness/erythema, blisters/vesicles, dry and scaly skin, and swollen skin areas [46, 48]. Wittkowski et al. [50] used the IPQ-R to investigate symptoms and emphasize that itching is the most frequently mentioned symptom attributed to atopic dermatitis in their sample: 98.6% of their sample believed that itchiness is related to atopic dermatitis. Further reported symptoms are sleep disorders (66.2%), pain (57.7%), sore eyes (49.6%), and fatigue (36.3%). Mollerup et al. [49] showed that women are significantly more likely to report itching and fatigue than men. Two studies [51, 52] used the B-IPQ [27] to investigate cognitive illness representations. Mean values of the identity dimensions range from 4.57 (SD=3.32) in a sample of patients with contact dermatitis (including ACD and ICD) [51] to 6.77 (SD=1.90) in patients with AD [52] indicating that patients perceive their illness as highly symptomatic, which implies a strong illness identity. Values are presented in detail in Table 2 and graphically in Fig. 4.

**Table 2:** Mean values of the B-IPQ and IPQ-R scales

	Benyamini et al. [51]				Březinová et al. [52]	Wittkowski et al. [50]
IPQ type	B-IPQ Scale range, 0-10				B-IPQ Scale range, 0-10	IPQ-R Scale range, 0-5
Diagnosis	Total sample	CD (incl. ACD and ICD)	AD (acc. to Simpson and Hanifin [53])	OD (acc. to Mathias’s criteria [54])	AD (acc. to Hanifin-Rajka’s criteria [55])	AD
Sample size	221	101	66	54	128	284
**Identity**	5.17 (SD=3.27)	4.57 (SD=3.32)	5.67 (SD=2.97)	5.67 (SD=3.41)	6.77 (SD=1.90)	
**Timeline**	5.66 (SD=3.25)	4.96 (SD=3.42)	6.28 (SD=2.83)	6.21 (SD=3.22)	8.06 (SD=2.09)	
Acute/chronic						3.93 (SD=0.81)
Cyclic						3.54 (SD=0.77)
**Consequences**	5.44 (SD=3.26)	4.82 (SD=3.46)	5.82 (SD=2.68)	6.08 (SD=3.42)	6.73 (SD=2.50)	2.82 (SD=1.05)
**Potential for cure/control**						
Personal control	3.54 (SD=3.24)	3.80 (SD=3.50)	3.22 (SD=2.55)	3.47 (SD=3.50)	5.79 (SD=2.24)	3.57 (SD=0.76)
Treatment control	5.89 (SD=3.06)	6.08 (SD=3.16)	6.46 (SD=2.49)	4.76 (SD=3.30)	6.80 (SD=2.45)	3.33 (SD=0.76)
**Coherence**	4.95 (SD=3.63)	4.93 (SD=3.72)	3.92 (SD=3.30)	6.29 (SD=3.49)	6.66 (SD=2.21)	2.79 (SD=1.06)
**Emotional representation**						2.97 (SD=1.01)
Emotional effect	4.96 (SD=3.50)	4.37 (SD=3.63)	5.24 (SD=3.22)	5.70 (SD=3.46)	6.30 (SD=2.90)	
Concern	6.68 (SD=3.13)	6.05 (SD=3.33)	7.10 (SD=2.66)	7.35 (SD=3.12)	7.59 (SD=2.30)	
Overall score					57.99 (SD=10.83)	
**Cause**						
Immunity causes						2.68 (SD=0.78)
Psychological causes						2.97 (SD=0.87)
Risk causes						2.61 (SD=0.62)
Chance causes						2.08 (SD=0.84)

*ACD* allergic contact dermatitis, *AD* atopic dermatitis, *B-IPQ* Brief Illness Perception Questionnaire, *CD* contact dermatitis, *ICD* irritant/irritative contact dermatitis, *OD* occupational dermatitis, *IPQ-R* revised Illness Perception Questionnaire

### Cause

All studies report suspected causes of the skin diseases. These can be classified into endogenous and exogenous causes (see Table 3). The diseases are often attributed to allergen exposure. This assumption remains unchanged, even if a patch test does not reveal sensitization [48]. In case of confirmed sensitization, effective allergen avoidance at workplace often seems impossible because of co-workers’ behaviors (e.g., contaminate work areas such as door handles) [48]. Furthermore, studies show that psychological factors (e.g., stress) are perceived as key causal factors [49–52]. Statements regarding the work-relatedness of the disease varied. While some participants identified certain causative agents in their workplace, other participants were unable to identify any cause and were more likely to suspect causes outside of work. One study showed that occupational chemicals, which are labeled as environmentally friendly, sometimes are not perceived as skin irritating [47]. Two studies also reported an uncertainty regarding the cause of the disease as well as inability to name causes [47, 51]. Bathe et al. [46] further reported that only a few participants acknowledge personal responsibility for their skin disease.

**Table 3:** Suspected causes of eczematous skin diseases

**Endogenous factors**	*Psychological factors*, e.g., mood, mental, or emotional state; stress; overwork; anxiety and anger; family problems
	*Medical conditions*, such as hormonal changes, weak immune system (e.g., infections, cold, influenza, fever); genetic disposition or heredity
**Exogenous factors**	*Behavioral factors:* frequent hand washing (incl. use of soap or hygiene products); nutrition or consumption of alcohol (e.g., red wine) or certain foods (e.g., pork); usage of emollients
	*Environmental factors:* climatic conditions (e.g., heat, change of climate, sun exposure); exposure to allergens (e.g., ingredients in cosmetics or emollients)
	*Domestic factors:* contact to household chemicals (e.g., detergents or laundry products); handling of food
	*Occupational factors:* contact to irritants, chemicals or allergens (e.g., oils, paints and varnishes, detergents, hygiene and laundry products—sometimes indirectly by soiled door handles); wet work (incl. sweaty hands, especially while wearing protective gloves, handwashing); physical friction (e.g., while construction work, machine maintenance, or gardening); handling of food or plants

### Timeline

None of the qualitative studies reported data regarding the expected timeline of the disease. We found long timeline beliefs, especially in study groups with AD (see Table 2). Values vary between 6.28 (SD=2.83) [51] and 8.06 (SD=2.09) [52] for the B-IPQ question “How long do you think your illness will continue?” (Scale: 0 = “a very short time”; 10 = “forever”). These results indicate a perceived chronic course of the disease. Results of Wittkowski et al. [50] underpin this assumption, as they found increased values on the bipolar timeline scale (acute/chronic) of the IPQ-R: M=3.93 (SD=0.81; scale range, 1-5). In addition, cyclical timeline perceptions are reported for AD patients (IPQ-R timeline scale (cyclical) M=3.54, SD=0.77) [50].

### Consequences

**Fig. 5:**
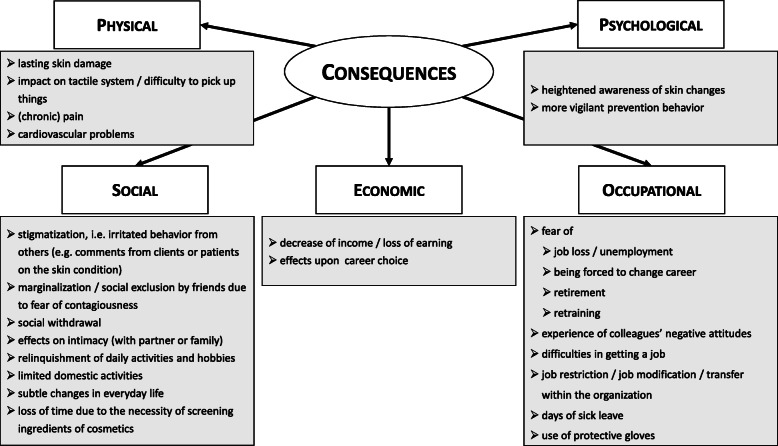
Expected and perceived consequences in terms of several dimensions. Summary of data from qualitative studies [46–48] and Mollerup et al. [49]

Perceived and experienced consequences—extracted from the qualitative studies [46–48] and Mollerup et al. [49]—are summarized in Fig. 5. Data can be classified into five sub-categories. It should be noted that these are in an interdependent relationship. Certain aspects fit into different categories; hence, these were assigned to the most appropriate one.

Benyamini et al. [51] and Březinová et al. [52] report heterogeneous data of the consequences scale of the B-IPQ, ranging from 4.82 (SD=3.46) in a group of CD patients to 6.73 (SD=2.50) in AD patients each with large standard deviations (see Table 2 and Fig. 4). Overall lower expected consequences are reported by Wittkowski et al. [50] although the analysis showed differences within their study insofar as members of the National Eczema Society reported significantly more consequences than the comparison group of students (IPQ-R consequence scale: M=3.62 (SD=0.84) vs. 2.19 (SD=0.74); *p* < 0.001) [50].

### Personal and treatment control

Study participants of the qualitative studies described different approaches to maintain control. While the IPQ-R and the B-IPQ distinguish between personal and treatment control, we assigned data from the qualitative studies [46–48] to the dimensions (1) working conditions, (2) skin protection, and (3) diagnostic procedures and treatment (see Table 4). Participants included in the qualitative studies additionally stated that following rules rigorously, self-acceptance as well as taking personal responsibility are pivotal to keep up control.

**Table 4:** Perceived controllability: Summary of data from qualitative studies [46–48]

**(1) Working conditions** *Improving/controlling skin disease through …*	… transferring to workplace with less or without exposure to irritants or allergens … avoidance or change of irritants in the workplace … being informed about hazard substances … reduction of working hours … time off work (sick leave or holiday) … change of company or profession … retraining
**(2) Skin protection** *Improving/controlling skin disease through …*	… usage of skin protection measures (e.g., protection gloves, cotton gloves, and emollients) *Certain barriers seem to limit the use of these measures: usage is experienced as problematic* (e.g., *emollients lead to greasy and slippery hands*); *misconceptions about the effect of emollients* (e.g., *assumption that effect gradually diminishes*); *negative outcome expectancies* (e.g., *creams provoke eczema*)
**(3) Diagnostic procedures and treatment** *Improving/controlling skin disease through …*	… increased usage of diagnostic procedures *Clarification of the cause of the disease by means of test schedules* (e.g., *patch tests*) *seems to be important for those affected in order to objectify the causes of the disease and thus gain a higher degree of control over the disease.* … self-medication (e.g., household remedies, alternative therapies) *Self-medication is often performed due to dissatisfaction with the dermatological treatment attempts.*

Benyamini et al. [51] found rather low perceived personal control across all groups included in their study with lowest amount of personal control in patients with AD: B-IPQ: 3.22 (SD = 2.55). In comparison, Březinová et al. [52] report a moderately better personal control (B-IPQ: 5.79; SD = 2.24). Except patients with OD (B-IPQ: 4.76; SD = 3.30), high treatment control beliefs were found among all study groups with values varying between 6.08 (SD = 3.16) in patients with CD and 6.80 (SD = 2.45) in patients with AD (see Table 2).

### Coherence

None of the qualitative studies provides data on how well the disease is understood. Benyamini et al. [51] report low values respecting coherence in patients with AD (M=3.92; SD=3.30; B-IPQ question “How well do you feel you understand your illness?”; scale: 0 = “don’t understand at all”; 10 = “understand very clearly”). While patients with CD also have slightly good understanding of their disease (M=4.95; SD=3.63), OD patients have an even better understanding of their disease: M=6.29 (SD= 3.49) [51]. In contrast to that, results of Březinová et al. [52] show the highest value on the coherence item for AD patients (M=6.66; SD=2.21).

### Emotional representations

Except one, all studies report results related to emotional representations. Mean values of the B-IPQ scale indicate strong emotional effect with scores of up to 6.30 (SD=2.90) in patients with AD [52] and 5.70 (SD=3.46) in patients with occupational dermatitis [51] (see Table 2 and Fig. 4). Wittkowski et al. [50] report higher values among members of the National Eczema Society and significant lower values among the student study group (IPQ-R emotional representation scale: M=3.60 (SD=0.90) vs. 2.48 (SD=0.79); *p* < 0.001). In comparison, feelings of depression, upset, anger, and anxiety are less common among students. Extracted data from the qualitative studies [46–48], in addition reveal complex emotional representations in patients with eczematous skin diseases. We found marked impairment of emotional well-being of participants, describing feelings of shame, dejection, helplessness, apathy, feelings of indolence as well as feelings of agitation. Participants also reported feelings of stigmatization and stress, especially during social interaction in times of severe eczema or when wearing cotton gloves. This causes frustration because ones seem to be different from others. Reported insecurity and anxiety about the future are also been assigned to emotional representations. Therefore, specific fears, such as worry about job loss or itching, were reported in the studies reviewed. And study participants described emotional strain and feeling guilty toward the employer due to sick leave [46–48]. These findings are in line with those from Benyamini et al. [51] and Březinová et al. [52], who report numerous concerns in patients with occupational dermatitis (M=7.35; SD=3.12) and especially atopic dermatitis (M=7.59; SD=2.30).

### Triangulation

We explored relationships within studies and between studies regarding levels of convergence, complementarity, and discrepancy for each of the reported dimensions of the CSM (see Additional file 5).

### Quality assessment

We used the Mixed Methods Appraisal Tool (MMAT) [44, 45] to assess the quality of studies included in this review. Additional file 6 presents the quality assessment for each study in detail. All studies affirm the two screening questions of the tool. The qualitative studies seem to be at a good quality level. To assess representativeness in quantitative studies, we checked indicators, such as description of the target group, inclusion and exclusion criteria and reasons for non-participation. However, some studies do not report appropriate information to judge this criterion. This extends to judgment of nonresponse bias, too. Nevertheless, it must be borne in mind that low reporting quality not necessarily indicates low study quality.

## Discussion

The aim of this systematic mixed studies review was to provide an overview of the representations of health and illness of patients with eczematous skin diseases. The narrative synthesis of the seven studies that met the inclusion criteria demonstrated that patients with ESD form their own illness perceptions. Besides strong illness identity and long timeline beliefs, we identified various suspected causes for the disease from the studies. Our results furthermore indicate that patients expect severe consequences in various contexts (e.g., social, economic, and occupational) and perceive a strong emotional impact of the skin disease.

As pointed out before, we refer to atopic dermatitis and contact dermatitis as eczematous skin diseases. This distinction, however, is only of limited appropriateness for these complex diseases, since AD is primarily an endogenous disease; it often manifests during childhood and usually has a relapsing course. If there is no remission during childhood or if there is a relapse in adolescence, the disease can occur continuously and may have a chronic course. AD is also frequently associated with other atopic diseases such as allergic asthma and allergic rhinitis [1, 2]. In contrast, exogenous causes can induce CD. Exposure to irritants may lead to irritant CD, which accounts for about 80% of all CD cases. Allergic contact dermatitis occurs after prior sensitization upon exposure to a specific contact allergen. In either instance, avoidance of exposure to allergens and irritants is of particular importance to prevent CD becoming a chronic condition [5, 6]. Because of the difficulty of clinically differentiating ICD and ACD and due to the fact that both may co-exist [5], both diagnoses have been included in the present review. Furthermore, AD and CD may have similar impacts on the lives of the affected individuals, in particular regarding impairment of quality of life, occupational impact, and higher risk for anxiety and depression [7, 8, 10, 12–16]. When examining the emotional representations in the present review, a key finding was that studies report a strong emotional effect and numerous concerns in nearly all study groups regardless of whether the diagnosis was CD or AD. These findings are consistent with a recent published study including AD patients across nine European countries on emotional consequences [58], which demonstrated that nearly 57% are emotionally burdened due to living with AD. Broadbent et al. [26] have also demonstrated the association of stronger emotional representations with reduced quality of life and higher depression and anxiety, which supports these results.

Despite the heterogeneity of our studies, triangulation revealed strong illness identity among the study participants, in particular patients with AD, meaning that these patients view their illness as highly symptomatic. In their meta-analytic review, Hagger and Orbell [25] investigated intercorrelations between the dimensions of the CSM across various studies and revealed a logical structure of relationships between the cognitive dimensions. They, for example, found positive correlations between illness identity, timeline, and consequences. Furthermore, identity is significant negatively associated to perceived control. In practice, this could imply that participants with a strong illness identity perceive their disease as more chronic, less controllable, and associate their disease with more serious consequences. However, it has to be considered that Hagger and Orbell [25] report statistical analyses to determine construct and discriminant validity of the model that do not reveal causal relationships (e.g., if patients perceive more serious consequences because of a highly symptomatic disease or vice versa). Since AD being a primarily endogenous and occasionally chronic disease and, as found in our review, is perceived as highly symptomatic (e.g., due to itching), future studies should concentrate on the investigation of illness representations considering different levels of disease severity. In terms of coping behavior, relationships between consequences and identity to maladaptive coping, namely “avoidance/denial” and “expressing emotions,” have been shown [25]. The same authors [25], furthermore, revealed a strong negative association of consequences and identity to the adaptive illness outcome “physical functioning” and positive association to “psychological distress.”

In our review, we assigned the reported causes to two main categories (endogenous and exogenous causes) and six subcategories, namely, psychological factors, medical condition, behavioral factors, environmental factors, domestic factors, and occupational factors. Moss-Morris et al. [40] carried out analyses on the causal items of the IPQ-R, and identified an underlying four-factorial structure of the scale, comprising psychological attributions, risk factors, immunity, and accident or chance. This structure, however, could only be reproduced to a limited extent by Wittkowski et al. [59] in a study with AD patients. In their meta-analytic review, Hagger and Orbell [25] also list various categories that can be used to classify the causes: biological causes, emotional causes, environmental causes, and psychological causes. They consider that categories might overlap, which impedes the interpretation of results [25]. The classification of perceived causes in the present review (see Table 3) is largely congruent with the subscales formulated by Moss-Morris et al. [40] and categories of Hagger and Orbell [25]. However, behavior-related causes (e.g., frequent hand washing, use of emollients) could only be assigned to a category or subscale to a limited extent. They fit best into the subscale “risk factors” postulated by Moss-Morris [40] (cf. item: “My own behavior”), although this item was originally assigned to the subscale “psychological attributions.” Future studies should hence consider a disease-specific adaptation of the cause-scale already during study planning.

Interestingly, we identified several perceived causes in the included studies, which are not represented in the relevant literature, namely, the consumption of red wine or pork. Although these causes cannot be ruled out in individual cases, plausible associations are seldom reported in clinical trials and tend to be overestimated, e.g., in terms of the relationship of hand eczema and alcohol consumption [60, 61]. Our review in addition revealed the widespread assumption that emollients or the ingredients of emollients—despite negative patch test results—could be the causative agent of the skin disease. Although this assumption seems paradoxical at first glance, it has already been described in the literature for protective creams and emollients [62]. As direct application of emollients to inflamed skin is often poorly tolerated, current guidelines, e.g., for the treatment of AD, recommend treatment of acute flare-ups first [63]. It is of particular importance for clinical practice and health care professionals to consider this, because—against the backdrop of the CSM—this example highlights the close connection between illness perceptions and the shown coping behavior (e.g., adherence to treatment recommendations). In practice, health care professionals should be aware of the individualized perceptions of patients, which may not always be consistent with medical facts [20, 22]. Furthermore, Leventhal et al. [23] stated that patients may conceal their reasons for non-adherence in order to avoid conflicts between their own perceptions and the underlying concept of the health care professional.

### Strengths and limitations

This review followed a pre-defined methodological procedure regarding the identification and analysis of the studies [33]. It was however necessary to deviate from the protocol because it was not possible to recruit information specialists for planning and peer reviewing the search strategies. This may occasionally lead to deficient search strategies (e.g., in terms of precision or sensitivity), and impair the overall quality of our review [64]. Due to the heterogeneity of the included studies, we carried out narrative, primarily descriptive synthesis and refrained from applying of meta-analytic methods, a reason why we are unable to provide further information on underlying processes or interrelationships. As noted in several reviews, the generalizability of results may be limited due to publication bias since negative (non-significant) results may not have been published and consequently may have been missed by systematic searches. This may influence the results of the present review. Finally, it has to be considered that all studies, except one [50], obtained data in clinical contexts. This may have led to an overestimation of illness perceptions and further outcomes as the participants may have had higher disease burden compared to those outside these settings or not under medical treatment, respectively (selection bias). Nevertheless, conducting a systematic mixed studies review for this topic, which involves an integration of studies and synthesis methods, is a major strength of this review since this approach enables a better understanding of complex phenomena [65, 66].

## Conclusion

In summary, our review suggests that patients with eczematous skin diseases have complex illness representations regarding their disease as described by Leventhal et al. [23] in the Common Sense Model. These representations interrelate with the coping behavior and crucial illness outcomes. It seems to be important for medical professionals to consider them during treatment and counseling to ensure patient-centered care.

## Supplementary Information

**Additional file 1.** PRISMA checklist.

**Additional file 2.** Full electronic search strategies.

**Additional file 3.** List of excluded studies with explanations.

**Additional file 4.** Inclusion and exclusion criteria in tabular form.

**Additional file 5.** Results of the Triangulation.

**Additional file 6.** Quality Assessment.

## Data Availability

Most of the data analyzed in this review are included in the published article and its supplementary information files. Further datasets used and analyzed during the current review are available from the corresponding author on reasonable request.

## Abbreviations

A&ICD: Allergic and irritant contact dermatitis
ACD: Allergic contact dermatitis
AD: Atopic dermatitis
AE: Atopic eczema
AHE: Allergic hand eczema
B-IPQ: Brief Illness Perception Questionnaire
CD: Contact dermatitis
ESD: Eczematous skin diseases
CSM: Common Sense Model of Self-Regulation of Health and Illness
ECD: Endogenous contact dermatitis
HE: Hand eczema
I&ATCD: Irritant and atopic contact dermatitis
ICD: Irritant/irritative contact dermatitis
IHE: Irritant/irritative hand eczema
IPQ: Illness Perception Questionnaire
IPQ-R: Revised Illness Perception Questionnaire
MMAT: Mixed Methods Appraisal Tool
NES: National Eczema Society
NOSQ-2002: Nordic Occupational Skin Questionnaire
OD: Occupational dermatitis
PRISMA: Preferred Reporting Items for Systematic Reviews and Meta-Analyses
QoL: Quality of life
UK: United Kingdom
