# Supplementary material for: Illness perceptions of adults with eczematous skin diseases: a systematic mixed studies review
Source: Syst Rev. 2021 May 7;10:141. doi: 10.1186/s13643-021-01687-5 (PMC8106167; doi:10.1186/s13643-021-01687-5)
Supplement: Supplementary file 2 — Additional file 2. Full electronic search strategies. [file 13643_2021_1687_MOESM2_ESM.docx]

**Additional file 2 – Database search strategies**

1 Medline via PubMed 2

2 The Cochrane Library 3

3 PsycInfo via EBSCO host 4

4 PSYNDEX via EBSCO host 5

5 CINAHL via EBSCO host 6

6 Web of Science 7

7 Scopus 8

| **Search results for each database** | |
| --- | --- |
| Medline via PubMed | 2.413 |
| The Cochrane Library | 343 |
| PsycInfo via EBSCO host | 719 |
| PSYNDEX via EBSCO host | 344 |
| CINAHL via EBSCO host | 185 |
| Web of Science Core Collection | 1.340 |
| Scopus | 1.391 |
|  | **6.735** |

# Medline via PubMed

Date of search: February 20, 2019

| **Search** | **Query** | **Items found** |
| --- | --- | --- |
| [#1](https://www.ncbi.nlm.nih.gov/pubmed) | occupational dermatitis | 11.314 |
| [#2](https://www.ncbi.nlm.nih.gov/pubmed) | allergic contact dermatitis | 16.448 |
| [#3](https://www.ncbi.nlm.nih.gov/pubmed) | irritant contact dermatitis | 5.028 |
| [#4](https://www.ncbi.nlm.nih.gov/pubmed) | atopic dermatitis | 25.674 |
| [#5](https://www.ncbi.nlm.nih.gov/pubmed) | contact dermatitis | 38.069 |
| [#6](https://www.ncbi.nlm.nih.gov/pubmed) | dermatitis | 117.559 |
| [#7](https://www.ncbi.nlm.nih.gov/pubmed) | dermatos* | 36.749 |
| [#8](https://www.ncbi.nlm.nih.gov/pubmed) | eczema* | 22.113 |
| [#9](https://www.ncbi.nlm.nih.gov/pubmed) | hand dermatoses | 7.683 |
| [#10](https://www.ncbi.nlm.nih.gov/pubmed) | (#1 OR #2 OR #3 OR #4 OR #5 OR #6 OR #7 OR #8 OR #9) | 148.031 |
| [#11](https://www.ncbi.nlm.nih.gov/pubmed) | illness representations | 1.157 |
| [#12](https://www.ncbi.nlm.nih.gov/pubmed) | illness perceptions | 19.241 |
| [#13](https://www.ncbi.nlm.nih.gov/pubmed) | health perceptions | 137.048 |
| [#14](https://www.ncbi.nlm.nih.gov/pubmed) | illness beliefs | 17.304 |
| [#15](https://www.ncbi.nlm.nih.gov/pubmed) | health beliefs | 182.837 |
| [#16](https://www.ncbi.nlm.nih.gov/pubmed) | illness cognitions | 16.330 |
| [#17](https://www.ncbi.nlm.nih.gov/pubmed) | illness theories | 1.616 |
| [#18](https://www.ncbi.nlm.nih.gov/pubmed) | lay concepts of health | 307 |
| [#19](https://www.ncbi.nlm.nih.gov/pubmed) | Attitude to Health | 500.988 |
| [#20](https://www.ncbi.nlm.nih.gov/pubmed) | Common sense model | 2.505 |
| [#21](https://www.ncbi.nlm.nih.gov/pubmed) | Health Knowledge, Attitudes, Practice[Mesh] | 101.044 |
| [#22](https://www.ncbi.nlm.nih.gov/pubmed) | Self Concept[Mesh] | 102.510 |
| [#23](https://www.ncbi.nlm.nih.gov/pubmed) | Attitude[Mesh] | 532.731 |
| [#24](https://www.ncbi.nlm.nih.gov/pubmed) | Self-Control[Mesh] | 1.601 |
| [#25](https://www.ncbi.nlm.nih.gov/pubmed) | Perception[Mesh] | 404.699 |
| [#26](https://www.ncbi.nlm.nih.gov/pubmed) | Health Behavior[Mesh] | 291.685 |
| #27 | (#11 OR #12 OR #13 OR #14 OR #15 OR #16 OR #17 OR #18 OR #19 OR #20 OR #21 OR #22 OR #23 OR #24 OR #25 OR #26) | 1.247.699 |
| **#28** | **(#10 AND #27)** | **2413** |

# The Cochrane Library

Date of search: February 20, 2019

| **Search** | **Query** | **Items found** |
| --- | --- | --- |
| #1 | (occupational dermatitis):ti,ab,kw (Word variations have been searched) | 139 |
| #2 | allergic contact dermatitis | 456 |
| #3 | irritant contact dermatitis | 274 |
| #4 | atopic dermatitis | 3.126 |
| #5 | contact dermatitis | 1.360 |
| #6 | dermatitis | 6.409 |
| #7 | dermatos* | 1.294 |
| #8 | eczema* | 2.917 |
| #9 | hand dermatoses | 273 |
| #10 | #1 OR #2 OR #3 OR #4 OR #5 OR #6 OR #7 OR #8 OR #9 | 8.714 |
| #11 | illness representations | 112 |
| #12 | illness perceptions | 745 |
| #13 | health perceptions | 3.434 |
| #14 | illness beliefs | 679 |
| #15 | health beliefs | 2.739 |
| #16 | illness cognitions | 159 |
| #17 | illness theories | 246 |
| #18 | lay concepts of health | 64 |
| #19 | Attitude to Health | 9.095 |
| #20 | Common sense model | 931 |
| #21 | MeSH descriptor: [Health Knowledge, Attitudes, Practice] explode all trees | 5.396 |
| #22 | MeSH descriptor: [Self Concept] explode all trees | 6.407 |
| #23 | MeSH descriptor: [Attitude] explode all trees | 34.854 |
| #24 | MeSH descriptor: [Self-Control] explode all trees | 157 |
| #25 | MeSH descriptor: [Perception] explode all trees | 16.825 |
| #26 | MeSH descriptor: [Health Behavior] explode all trees | 32.406 |
| #27 | #11 OR #12 OR #13 OR #14 OR #15 OR #16 OR #17 OR #18 OR #19 OR #20 OR #21 OR #22 OR #23 OR #24 OR #25 OR #26 | 66.574 |
| **#28** | **#10 AND #27** | **343** |

# PsycInfo via EBSCO host

Date of search: February 20, 2019

| **Search** | **Query** | **Items found** |
| --- | --- | --- |
| S1 | occupational dermatitis | 41 |
| S2 | allergic contact dermatitis | 31 |
| S3 | irritant contact dermatitis | 2 |
| S4 | atopic dermatitis | 387 |
| S5 | contact dermatitis | 86 |
| S6 | dermatitis | 820 |
| S7 | dermatos* | 150 |
| S8 | eczema* | 430 |
| S9 | hand dermatoses | 13 |
| S10 | neuroderm* | 131 |
| S11 | DE "Skin Disorders" | 1.565 |
| S12 | DE "Allergic Skin Disorders" | 70 |
| S13 | DE "Work Related Illnesses" | 1.022 |
| S14 | S1 OR S2 OR S3 OR S4 OR S5 OR S6 OR S7 OR S8 OR S9 OR S10 OR S11 OR S12 OR S13 | 3.742 |
| S15 | DE "Health Knowledge" | 7.272 |
| S16 | DE "Health Attitudes" | 9.837 |
| S17 | DE "Attitudes" | 25.516 |
| S18 | DE "Physical Illness (Attitudes Toward)" | 2.399 |
| S19 | DE "Coping Behavior" | 45.502 |
| S20 | DE "Behavior" | 32.238 |
| S21 | DE "Illness Behavior" | 3.579 |
| S22 | MA Health Knowledge, Attitudes, Practice | 24.341 |
| S23 | MA Self Concept | 29.149 |
| S24 | MA Attitude | 83.522 |
| S25 | MA Self-Control | 438 |
| S26 | MA Perception | 120.302 |
| S27 | MA Health Behavior | 41.140 |
| S28 | illness perception* | 19.826 |
| S29 | illness representation* | 2.790 |
| S30 | health perception* | 94.706 |
| S31 | illness belief* | 9.752 |
| S32 | health belief* | 43.766 |
| S33 | illness cognition* | 15.498 |
| S34 | illness theor* | 21.014 |
| S35 | lay concepts of health | 763 |
| S36 | Attitude to Health | 188.935 |
| S37 | Common sense model | 1.752 |
| S38 | S15 OR S16 OR S17 OR S18 OR S19 OR S20 OR S21 OR S22 OR S23 OR S24 OR S25 OR S26 OR S27 OR S28 OR S29 OR S30 OR S31 OR S32 OR S33 OR S34 OR S35 OR S36 OR S37 | 547.984 |
| **S39** | **S14 AND S38** | **719** |

# PSYNDEX via EBSCO host

Date of search: February 20, 2019

| **Search** | **Query** | **Items found** |
| --- | --- | --- |
| S1 | occupational dermatitis | 3 |
| S2 | allergic contact dermatitis | 1 |
| S3 | irritant contact dermatitis | 0 |
| S4 | atopic dermatitis | 141 |
| S5 | contact dermatitis | 9 |
| S6 | dermatitis | 220 |
| S7 | dermatos* | 29 |
| S8 | eczema* | 71 |
| S9 | hand dermatoses | 1 |
| S10 | neuroderm* | 345 |
| S11 | DE "Skin Disorders" | 373 |
| S12 | DE "Allergic Skin Disorders" | 35 |
| S13 | DE "Work Related Illnesses" | 494 |
| S14 | S1 OR S2 OR S3 OR S4 OR S5 OR S6 OR S7 OR S8 OR S9 OR S10 OR S11 OR S12 OR S13 | 1.226 |
| S15 | DE "Health Knowledge" | 405 |
| S16 | DE "Health Attitudes" | 1.046 |
| S17 | DE "Attitudes" | 2.512 |
| S18 | DE "Physical Illness (Attitudes Toward)" | 290 |
| S19 | DE "Coping Behavior" | 10.997 |
| S20 | DE "Behavior" | 1.761 |
| S21 | DE "Illness Behavior" | 1.434 |
| S22 | MA Health Knowledge, Attitudes, Practice | 49 |
| S23 | MA Self Concept | 3 |
| S24 | MA Attitude | 29 |
| S25 | MA Self-Control | 2 |
| S26 | MA Perception | 21 |
| S27 | MA Health Behavior | 4 |
| S28 | illness perception* | 1.000 |
| S29 | illness representation* | 163 |
| S30 | health perception* | 2.163 |
| S31 | illness belief* | 380 |
| S32 | health belief* | 841 |
| S33 | illness cognition* | 394 |
| S34 | illness theor* | 2.700 |
| S35 | lay concepts of health | 48 |
| S36 | Attitude to Health | 6.685 |
| S37 | Common sense model | 51 |
| S38 | S15 OR S16 OR S17 OR S18 OR S19 OR S20 OR S21 OR S22 OR S23 OR S24 OR S25 OR S26 OR S27 OR S28 OR S29 OR S30 OR S31 OR S32 OR S33 OR S34 OR S35 OR S36 OR S37 | 25.996 |
| **S39** | **S14 AND S38** | **344** |

# CINAHL via EBSCO host

Date of search: February 20, 2019

| **Search** | **Query** | **Items found** |
| --- | --- | --- |
| S1 | occupational dermatitis | 436 |
| S2 | allergic contact dermatitis | 563 |
| S3 | irritant contact dermatitis | 203 |
| S4 | atopic dermatitis | 3.635 |
| S5 | contact dermatitis | 2.001 |
| S6 | dermatitis | 10.056 |
| S7 | dermatos* | 1.651 |
| S8 | eczema* | 3.468 |
| S9 | hand dermatoses | 32 |
| S10 | neuroderm* | 16 |
| S11 | DE "Skin Disorders" | 5.959 |
| S12 | DE "Allergic Skin Disorders" | 2.817 |
| S13 | DE "Work Related Illnesses" | 4.430 |
| S14 | S1 OR S2 OR S3 OR S4 OR S5 OR S6 OR S7 OR S8 OR S9 OR S10 OR S11 OR S12 OR S13 | 13.668 |
| S15 | DE "Health Knowledge" | 24.430 |
| S16 | DE "Health Attitudes" | 87.134 |
| S17 | DE "Attitudes" | 3.226 |
| S18 | DE "Physical Illness (Attitudes Toward)" | 22.165 |
| S19 | DE "Coping Behavior" | 6.814 |
| S20 | DE "Behavior" | 16.772 |
| S21 | DE "Illness Behavior" | 12.605 |
| S22 | MA Health Knowledge, Attitudes, Practice | 13 |
| S23 | MA Self Concept | 21 |
| S24 | MA Attitude | 226 |
| S25 | MA Self-Control | 4 |
| S26 | MA Perception | 186 |
| S27 | MA Health Behavior | 146 |
| S28 | illness perception* | 8.765 |
| S29 | illness representation* | 1.164 |
| S30 | health perception* | 51.301 |
| S31 | illness belief* | 5.076 |
| S32 | health belief* | 29.572 |
| S33 | illness cognition* | 5.597 |
| S34 | illness theor* | 9.899 |
| S35 | lay concepts of health | 317 |
| S36 | Attitude to Health | 157.969 |
| S37 | Common sense model | 572 |
| S38 | S15 OR S16 OR S17 OR S18 OR S19 OR S20 OR S21 OR S22 OR S23 OR S24 OR S25 OR S26 OR S27 OR S28 OR S29 OR S30 OR S31 OR S32 OR S33 OR S34 OR S35 OR S36 OR S37 | 241.729 |
| **S39** | **S14 AND S38** | **185** |

# Web of Science

Date of search: February 20, 2019

| **Search** | **Query** | **Items found** |
| --- | --- | --- |
| #1 | TS=(occupational dermatitis) | 8.121 |
| #2 | TS=(allergic contact dermatitis) | 17.454 |
| #3 | TS=(irritant contact dermatitis) | 3.939 |
| #4 | TS=(atopic dermatitis) | 38.441 |
| #5 | TS=(contact dermatitis) | 31.817 |
| #6 | TS=(dermatitis) | 98.390 |
| #7 | TS=(dermatos*) | 44.986 |
| #8 | TS=(eczema*) | 25.185 |
| #9 | TS=(hand dermatoses) | 6.788 |
| #10 | #1 OR #2 OR #3 OR #4 OR #5 OR #6 OR #7 OR #8 OR #9 | 142.003 |
| #11 | TS=(illness representations) | 3.708 |
| #12 | TS=(illness perceptions) | 23.300 |
| #13 | TS=(health perceptions) | 128.199 |
| #14 | TS=(illness beliefs) | 16.044 |
| #15 | TS=(health beliefs) | 91.130 |
| #16 | TS=(illness cognitions) | 18.977 |
| #17 | TS=(illness theories) | 11.802 |
| #18 | TS=(lay concepts of health) | 1.638 |
| #19 | TS=(Attitude to Health) | 241.445 |
| #20 | TS=(Common sense model) | 20.735 |
| #21 | #11 OR #12 OR #13 OR #14 OR #15 OR #16 OR #17 OR #18 OR #19 OR #20 | 424.577 |
| **#22** | **#10 AND #21** | **1340** |

# Scopus

Date of search: February 22, 2019

| **Search** | **Query** | **Items found** |
| --- | --- | --- |
| [#1](https://www.ncbi.nlm.nih.gov/pubmed) | ALL("occupational dermatitis") | 3.212 |
| [#2](https://www.ncbi.nlm.nih.gov/pubmed) | ALL("allergic contact dermatitis") | 29.301 |
| [#3](https://www.ncbi.nlm.nih.gov/pubmed) | ALL("irritant contact dermatitis" ) | 5.516 |
| [#4](https://www.ncbi.nlm.nih.gov/pubmed) | ALL("atopic dermatitis" ) | 90.524 |
| [#5](https://www.ncbi.nlm.nih.gov/pubmed) | ALL("contact dermatitis") | 78.725 |
| [#6](https://www.ncbi.nlm.nih.gov/pubmed) | ALL("dermatitis") | 245.908 |
| [#7](https://www.ncbi.nlm.nih.gov/pubmed) | ALL(dermatos*) | 80.061 |
| [#8](https://www.ncbi.nlm.nih.gov/pubmed) | ALL( eczema*) | 84.495 |
| [#9](https://www.ncbi.nlm.nih.gov/pubmed) | ALL("hand dermatoses") | 6.905 |
| [#10](https://www.ncbi.nlm.nih.gov/pubmed) | #1 OR #2 OR #3 OR #4 OR #5 OR #6 OR #7 OR #8 OR #9 | 329.195 |
| [#11](https://www.ncbi.nlm.nih.gov/pubmed) | ALL("illness representations") | 4.900 |
| [#12](https://www.ncbi.nlm.nih.gov/pubmed) | ALL("illness perceptions") | 9.897 |
| [#13](https://www.ncbi.nlm.nih.gov/pubmed) | ALL("health perceptions") | 10.626 |
| [#14](https://www.ncbi.nlm.nih.gov/pubmed) | ALL("illness beliefs") | 3.174 |
| [#15](https://www.ncbi.nlm.nih.gov/pubmed) | ALL("health beliefs") | 45.716 |
| [#16](https://www.ncbi.nlm.nih.gov/pubmed) | ALL("illness cognitions") | 2.304 |
| [#17](https://www.ncbi.nlm.nih.gov/pubmed) | ALL("illness theories") | 1.173 |
| [#18](https://www.ncbi.nlm.nih.gov/pubmed) | ALL("lay concepts of health") | 50 |
| [#19](https://www.ncbi.nlm.nih.gov/pubmed) | ALL("Attitude to Health") | 147.365 |
| [#20](https://www.ncbi.nlm.nih.gov/pubmed) | ALL("Common sense model") | 3.001 |
| #21 | #11 OR #12 OR #13 OR #14 OR #15 OR #16 OR #17 OR #18 OR #19 OR #20 | 205.449 |
| **#22** | **#10 AND #21** | **1.391** |
