# Supplementary material for: Illness perceptions of adults with eczematous skin diseases: a systematic mixed studies review
Source: Syst Rev. 2021 May 7;10:141. doi: 10.1186/s13643-021-01687-5 (PMC8106167; doi:10.1186/s13643-021-01687-5)
Supplement: Supplementary file 3 — Additional file 3. List of excluded studies with explanations. [file 13643_2021_1687_MOESM3_ESM.docx]

**Additional file 3: List of excluded studies, listed by primary reason for exclusion**

**Participants <18 years included in sample**

Ahmed A, Shah R, Papadopoulos L, Bewley A. An ethnographic study into the psychological impact and adaptive mechanisms of living with hand eczema. Clin Exp Dermatol. 2015;40:495-501.

Ando T, Hashiro M, Noda K, Adachi J, Hosoya R, Kamide R, et al. Development and validation of the psychosomatic scale for atopic dermatitis in adults. J Dermatol. 2006;33:439-50.

Charman CR, Venn AJ, Ravenscroft JC, Williams HC. Translating Patient-Oriented Eczema Measure (POEM) scores into clinical practice by suggesting severity strata derived using anchor-based methods. Br J Dermatol. 2013;169:1326-32.

Evers AWM, Duller P, van de Kerkhof, P. C. M., van der Valk, P. G. M., de Jong, E. M. G. J., Gerritsen MJP, et al. The Impact of Chronic Skin Disease on Daily Life (ISDL): a generic and dermatology-specific health instrument. Br J Dermatol. 2008;158:101-8.

Fisker MH, Ebbehøj NE, Jungersted JM, Agner T. What do patients with occupational hand eczema know about skin care? Contact dermatitis. 2013;69:93-8.

Howells LM, Chalmers JR, Cowdell F, Ratib S, Santer M, Thomas KS. 'When it goes back to my normal I suppose': a qualitative study using online focus groups to explore perceptions of 'control' among people with eczema and parents of children with eczema in the UK. BMJ open. 2017;7:e017731.

Jung HJ, Bae JY, Kim JE, Na CH, Park GH, Bae YI, et al. Survey of disease awareness, treatment behavior and treatment satisfaction in patients with atopic dermatitis in Korea: A multicenter study; 2018;45:1172-80.

Kobyletzki LB von, Thomas KS, Schmitt J, Chalmers JR, Deckert S, Aoki V, et al. What Factors are Important to Patients when Assessing Treatment Response: An International Cross-sectional Survey. Acta Derm Venereol. 2017;97:86-90.

Long CC, Funnell CM, Collard R, Finlay AY. What do members of the national-eczema-society really want. Clin Exp Dermatol. 1993;18:516-22.

Lu Y, Duller P, van der Valk, P. G. M., Evers AWM. Helplessness as predictor of perceived stigmatization in patients with psoriasis and atopic dermatitis. Dermatology and Psychosomatics. 2003;4:146-50.

Magin PJ, Adams J, Heading GS, Pond DC, Smith W. Complementary and alternative medicine therapies in acne, psoriasis, and atopic eczema: results of a qualitative study of patients' experiences and perceptions. J Altern Complementary Med. 2006;12:451-7.

Magin P, Heading G, Adams J, Pond D. Sex and the skin: a qualitative study of patients with acne, psoriasis and atopic eczema. Psychol Health Med. 2010;15:454-62.

McAlister RO, Tofte SJ, Doyle JJ, Jackson A, Hanifin JM. Patient and physician perspectives vary on atopic dermatitis. Cutis. 2002;69:461-6.

Schmid-Ott G, Burchard R, Niederauer HH, Lamprecht F, Künsebeck HW. Stigmatization and quality of life of patients with psoriasis and atopic dermatitis. Hautarzt. 2003;54:852-7.

Schmid-Ott G, Kuensebeck HW, Jaeger B, Werfel T, Frahm K, Ruitman J, et al. Validity study for the stigmatization experience in atopic dermatitis and psoriatic patients. Acta Derm Venereol. 1999;79:443-7.

Torrelo A, Ortiz J, Alomar A, Ros S, ra, Prieto M, Cuervo J. Atopic dermatitis: impact on quality of life and patients' attitudes toward its management. Eur J Dermatol. 2012;22:97-105.

**Study did not report relevant data for the analysis**

Arcury TA, Qu, t SA, Mellen BG. An Exploratory Analysis of Occupational Skin Disease among Latino Migrant and Seasonal Farmworkers in North Carolina. J Agric Saf Health. 2003;9:221-32.

Arima K, Gupta S, Gadkari A, Hiragun T, Kono T, Katayama I, et al. Burden of atopic dermatitis in Japanese adults: Analysis of data from the 2013 National Health and Wellness Survey. J Dermatol. 2018;45:390-6.

Betlloch I, Izu R, Lleonart M, Ferrer M, Ferr, o J, Investigadores del estudio A. Attitude of the adult patient with atopic dermatitis to the disease and its treatment: the ACTIDA Study. Actas Dermosifiliogr. 2010;101:143-50.

Cvetkovski RS, Jensen H, Olsen J, Johansen JD, Agner T. Relation between patients' and physicians' severity assessment of occupational hand eczema. Br J Dermatol. 2005;153:596-600.

Dieris-Hirche J, Gieler U, Petrak F, Milch W, Te Wildt B, Dieris B, Herpertz S. Suicidal Ideation in Adult Patients with Atopic Dermatitis: A German Cross-sectional Study. Acta Derm Venereol. 2017;97:1189-95.

Dieris-Hirche J, Milch WE, Kupfer J, Leweke F, Gieler U. Atopic dermatitis, attachment and partnership: a psychodermatological case-control study of adult patients. Acta Derm Venereol. 2012;92:462-6.

Dorst J, Seikowski K. Skin, bonding and partnership in atopic dermatitis and psoriasis. Hautarzt. 2012;63:214-20.

Holness DL. Results of a quality of life questionnaire in a patch test clinic population. Contact dermatitis. 2001;44:80-4.

Jagou M, Bastuji-Garin S, Bourdon-Lanoy E, Penso-Assathiany D, Roujeau JC, Réseau d'Epidémiologie en D. Poor agreement between self-reported and dermatologists' diagnoses for five common dermatoses. Br J Dermatol. 2006;155:1006-12.

Leibovici V, Canetti L, Yahalomi S, Cooper-Kazaz R, Bonne O, Ingber A, Bachar E. Well being, psychopathology and coping strategies in psoriasis compared with atopic dermatitis: a controlled study. J. Eur Acad Dermatol Venereol. 2010;24:897-903.

Marron S, E. o, Tomas-Aragones L, Navarro-Lopez J, Gieler U, Kupfer J, et al. The psychosocial burden of hand eczema: Data from a European dermatological multicentre study. Contact dermatitis. 2018;78:406-12.

Meding B, Wrangsjö K, Järvholm B. Fifteen-year follow-up of hand eczema: persistence and consequences. Br J Dermatol. 2005;152:975-80.

Mizara A, Papadopoulos L, McBride SR. Core beliefs and psychological distress in patients with psoriasis and atopic eczema attending secondary care: the role of schemas in chronic skin disease. Br J Dermatol. 2012;166:986-93.

Qi He Leow M, Cao T. Perception on skin problems, skin care and knowledge needs of people in the Singapore community. Nurs J Singapore. 2018;45:20-6.

Schut C, Felsch A, Zick C, Hinsch KD, Gieler U, Kupfer J. Role of illness representations and coping in patients with atopic dermatitis: A cross-sectional study. J Eur Acad Dermatol Venereol. 2014;28:1566-71.

Seikowski K, Gelbrich M, Harth W. Sexual self-reflection in patients with atopic dermatitis and psoriasis. Hautarzt. 2008;59:297-303.

Silverberg JI, Gelf, J. M., Margolis DJ, Boguniewicz M, Fonacier L, et al. Patient burden and quality of life in atopic dermatitis in US adults: A population-based cross-sectional study. Ann Allergy Asthma Immunol. 2018;121:340-7.

Simpson EL, Bieber T, Eckert L, Wu R, Ardeleanu M, Graham NMH, et al. Patient burden of moderate to severe atopic dermatitis (AD): Insights from a phase 2b clinical trial of dupilumab in adults. J Am Acad Dermatol. 2016;74:491-8.

Stalder JF, Barbarot S, Wollenberg A, Holm EA, Raeve L de, Seidenari S, et al. Patient-Oriented SCORAD (PO-SCORAD): a new self-assessment scale in atopic dermatitis validated in Europe. Allergy. 2011;66:1114-21.

Stangier U, Barnhofer T, Aschoff S, Hoyer J. Predicting coping with atopic dermatitis and psoriasis. Zeitschrift für Gesundheitspsychologie. 1998;6:179-89.

Webers T. Neurodermitis: ärztliche Therapie oder Selbsthilfe? : Wie Patienten die ärztliche Therapie erleben, und warum Selbsthilfe bei der Krankheitsbewältigung hilft. Prävention. 1994;17:96-8.

Wei W, Anderson P, Gadkari A, Blackburn S, Moon R, Piercy J, et al. Extent and consequences of inadequate disease control among adults with a history of moderate to severe atopic dermatitis. J Dermatol. 2018;45:150-7.

Wei W, Anderson P, Gadkari A, Blackburn S, Moon R, Piercy J, et al. Discordance Between Physician- and Patient-Reported Disease Severity in Adults with Atopic Dermatitis: A US Cross-Sectional Survey. Am J Clin Dermatol. 2017;18:825-35.

Wittkowski A, Richards HL, Williams J, Main CJ. Factor analysis of the Revised Illness Perception Questionnaire in adults with atopic dermatitis. Psychol Health Med. 2008;13:346-59.

**Patients with other disease included in sample (e.g. psoriasis, asthma, hay fever, urticaria); data not separately analysed**

Diamond SF. Development of expertise in self-management of chronic illness: Narratives of older adults living with atopic dermatitis, Asthma, and allergies since childhood. Dissertation Abstracts International: Section B: The Sciences and Engineering. 2005;66:547.

Ergun M, Ermertcan AT, Ozturkcan S, Temeltas G, Deveci A, Dinc G. Sexual dysfunction in patients with chronic hand eczema in the Turkish population. J Sex Med. 2007;4:1684-90.

Holness DL, Nethercott JR. Is a worker's understanding of their diagnosis an important determinant of outcome in occupational contact dermatitis? Contact dermatitis. 1991;25:296-301.

Lazarov A, Rabin B, Fraidlin N, Abraham D. Medical and psychosocial outcome of patients with occupational contact dermatitis in Israel. J Eur Acad Dermatol Venereol. 2006;20:1061-5.

Magin PJ, Pond CD, Smith WT, Watson AB, Goode SM. Correlation and agreement of self-assessed and objective skin disease severity in a cross-sectional study of patients with acne, psoriasis, and atopic eczema. Int J Dermatol. 2011;50:1486-90.

Potocka A, Turczyn-Jabłońska K, Kieć-Swierczyńska M. Self-image and quality of life of dermatology patients. Int J Occup Med Environ Health. 2008;21:309-17.

Ring L, Kettis-Lindblad A, Kjellgren KI, Kindell Y, Maroti M, Serup J. Living with skin diseases and topical treatment: Patients' and providers' perspectives and priorities. J Dermatol Treat. 2007;18:209-18.

Verhoof EJA, Maurice-Stam H, Heymans HSA, Evers AWM, Grootenhuis MA. Psychosocial well-being in young adults with chronic illness since childhood: The role of illness cognitions. Child Adolesc Psychiatry Ment. Health. 2014;8.

**Diagnosis not medically confirmed**

Roosta N, Black DS, Peng D, Riley LW. Skin disease and stigma in emerging adulthood: impact on healthy development. J Cutan Med Surg. 2010;14:285-90.

**Conference abstract**

Schut C, Felsch A, Zick C, Hinsch K-D, Gieler U, Kupfer J. The importance of illness representations and coping for physical impairment in patients with atopic dermatitis: a 1-year-follow-up study. Acta Derm Venereol. 2016;96 Suppl 217:142.

**Full text of the study could not be retrieved**

Matos MM. Exploring individual disease burden in alopecia, atopic dermatitis, and psoriasis. Dissertation Abstracts International: Section B: The Sciences and Engineering. 2017;77(11-B)(E).

**Non-English language^[[1]](#footnote-1)^**

*Dutch*

Evers AWM, Duller P, van de Kerkhof, P. C. M., van der Valk, P. G. M., Gerritsen MJ, Otero ME, et al. The impact of chronic skin diseases on daily life: A disease-specific and generic assessment. Psychologie & Gezondheid. 2007;35:40-9.

*French*

Misery L. Burden of atopic dermatitis in adults. Ann Dermatol Venereol. 2017;144 Suppl 5:VS23-VS28.

*Japanese*

Okuno E, Agari I. Psychological stress responses in adult patients with atopic dermatitis. Japanese J. Health Psychol. 2002;15:49-58.

1. Due to the language barrier, we could not assess meeting of inclusion criteria. [↑](#footnote-ref-1)
