# Supplementary material for: Illness perceptions of adults with eczematous skin diseases: a systematic mixed studies review
Source: Syst Rev. 2021 May 7;10:141. doi: 10.1186/s13643-021-01687-5 (PMC8106167; doi:10.1186/s13643-021-01687-5)
Supplement: Supplementary file 4 — Additional file 4. Inclusion and exclusion criteria in tabular form. [file 13643_2021_1687_MOESM4_ESM.docx]

# Additional file 4 – Inclusion and exclusion criteria in tabular form

|  | **Inclusion criteria** | **Exclusion criteria** |
| --- | --- | --- |
| **Study design** | - quantitative studies - qualitative studies - mixed-method studies | - Editorials - Comments - Case reports - Systematic Reviews - Conference abstracts - Letters - Book chapters |
| **Language** | - include in review: English or German - provide in appendix: other languages |  |
| **Population** | - adults; >18 years - medically confirmed diagnosis of eczematous skin disease, e. g. atopic dermatitis, irritative contact dermatitis, allergic contact dermatitis or mixed diagnoses | - children, adolescents; <18 years - parents of affected children - medical professionals (e. g. physicians, nurses) - non-affected, healthy persons (including partners) - diagnosis of other chronic inflammatory skin disease (e. g. psoriasis, seborrheic dermatitis, rosacea, lupus erythematosus) |
| **Phenomenon of interest: Illness perceptions** | Study reports results assignable to at least one of Leventhal’s dimensions of the Common Sense Modell:   - **Identity**   assumptions about symptoms or the labels of the disease  (e. g. redness, itching, blisters)   - **Cause**   assumptions about the causes of the disease  (e. g. endogenous [genetic disposition] or exogenous causes [allergens, hazardous substances])   - **Timeline**   assumptions about the progression of the disease  (e. g. acute, chronic, cyclic)   - **Consequences**   assumptions about the consequences of the disease  (e. g. physical, psychological, social or economic consequences)   - **Control**   assumptions about the controllability of the disease  (e. g. personal control, treatment control) | Studies exclusively reporting:   - quality of life of patients with skin diseases - association of skin diseases and psychological disorders   (e. g. depression, anxiety)   - attitude to diagnostic procedures   (e. g. patch test, photochemotherapy) |
| **Context** | - all inpatient and outpatient health care settings |  |
