# Supplementary material for: Illness perceptions of adults with eczematous skin diseases: a systematic mixed studies review
Source: Syst Rev. 2021 May 7;10:141. doi: 10.1186/s13643-021-01687-5 (PMC8106167; doi:10.1186/s13643-021-01687-5)
Supplement: Supplementary file 5 — Additional file 5. Results of the Triangulation. [file 13643_2021_1687_MOESM5_ESM.docx]

**Additional File 5 – Triangulation**

1 Triangulation – Identity 2

2 Triangulation – Cause 3

3 Triangulation – Timeline 3

4 Triangulation – Consequences 4

5 Triangulation – Personal and Treatment Control 4

6 Triangulation – Coherence 5

7 Triangulation – Emotional Representation 5

# Triangulation – Identity

*
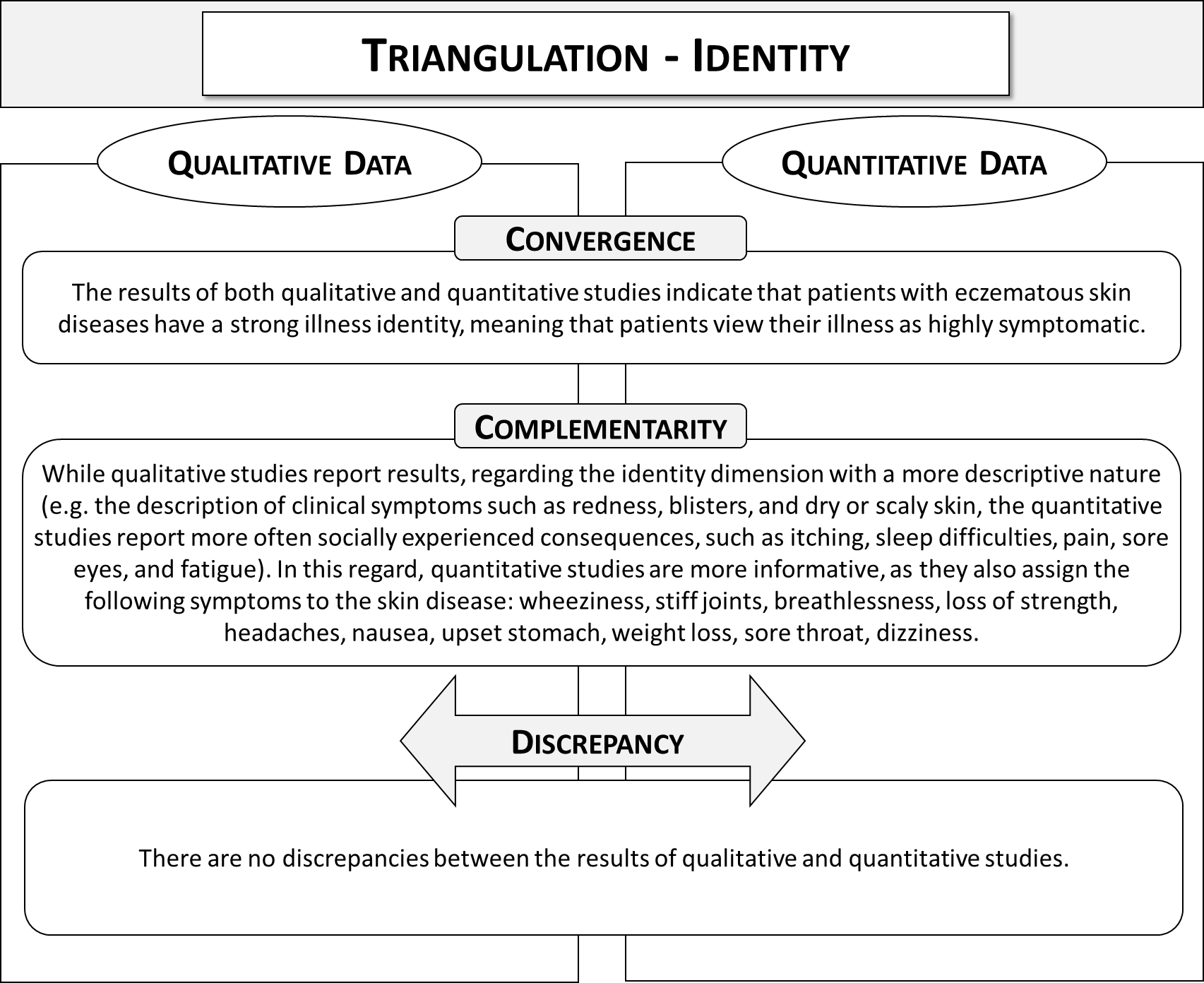
*

# Triangulation – Cause


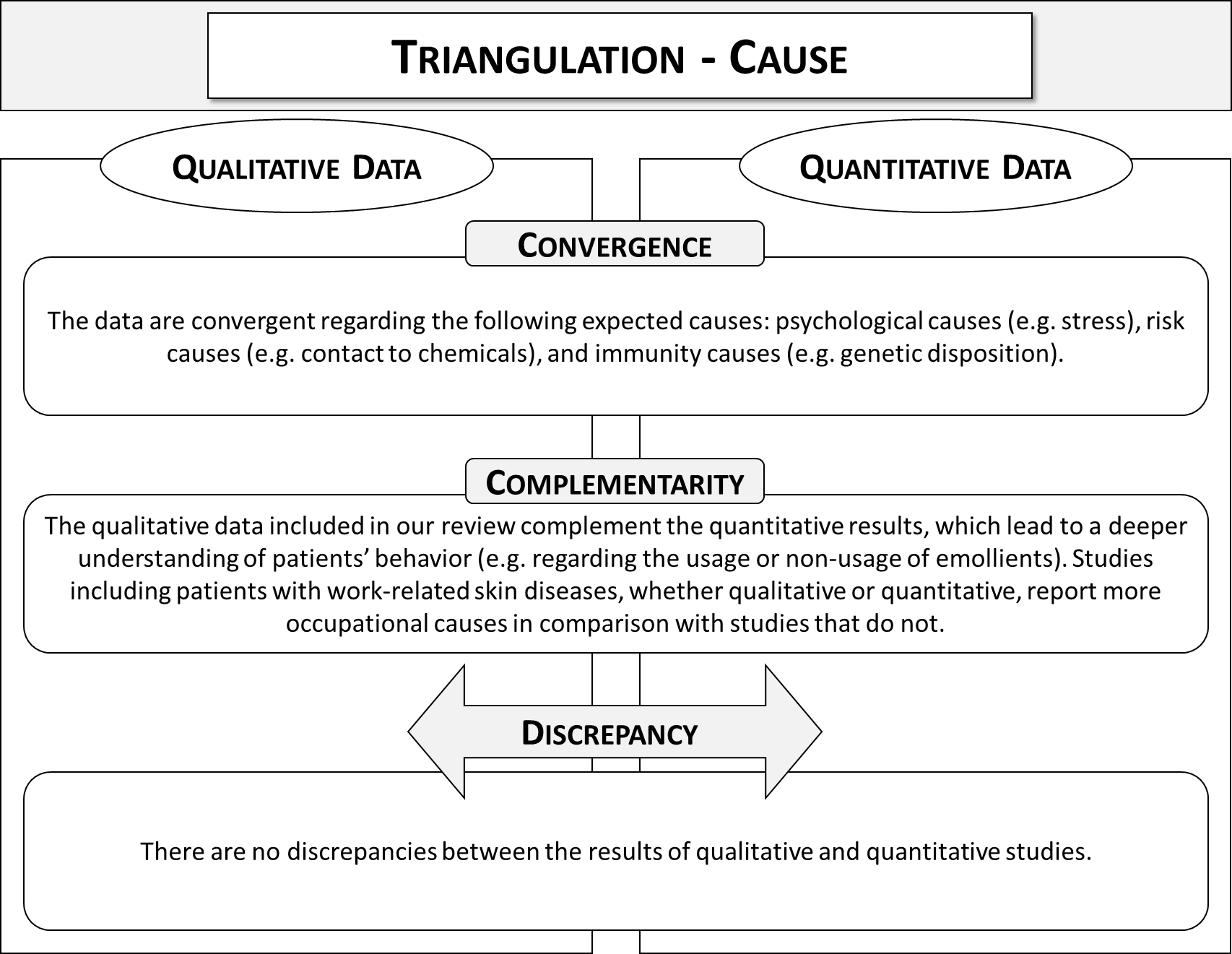


# Triangulation – Timeline


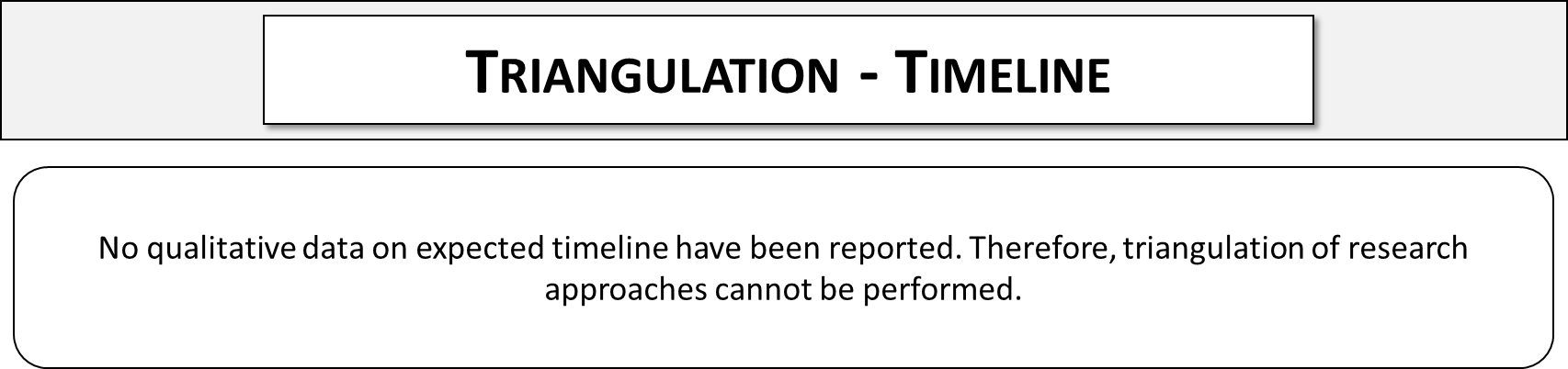


# Triangulation – Consequences

**
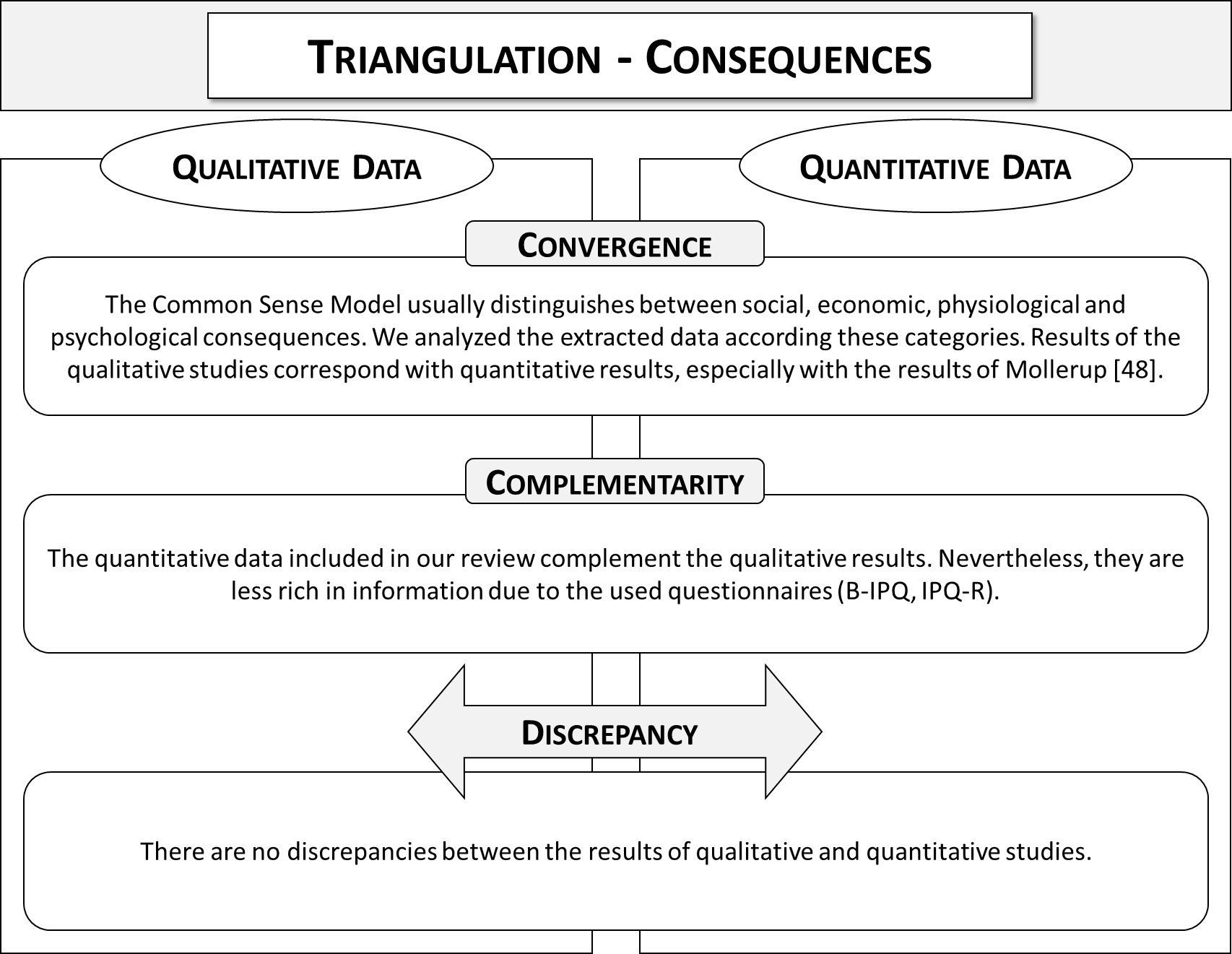
**

# Triangulation – Personal and Treatment Control


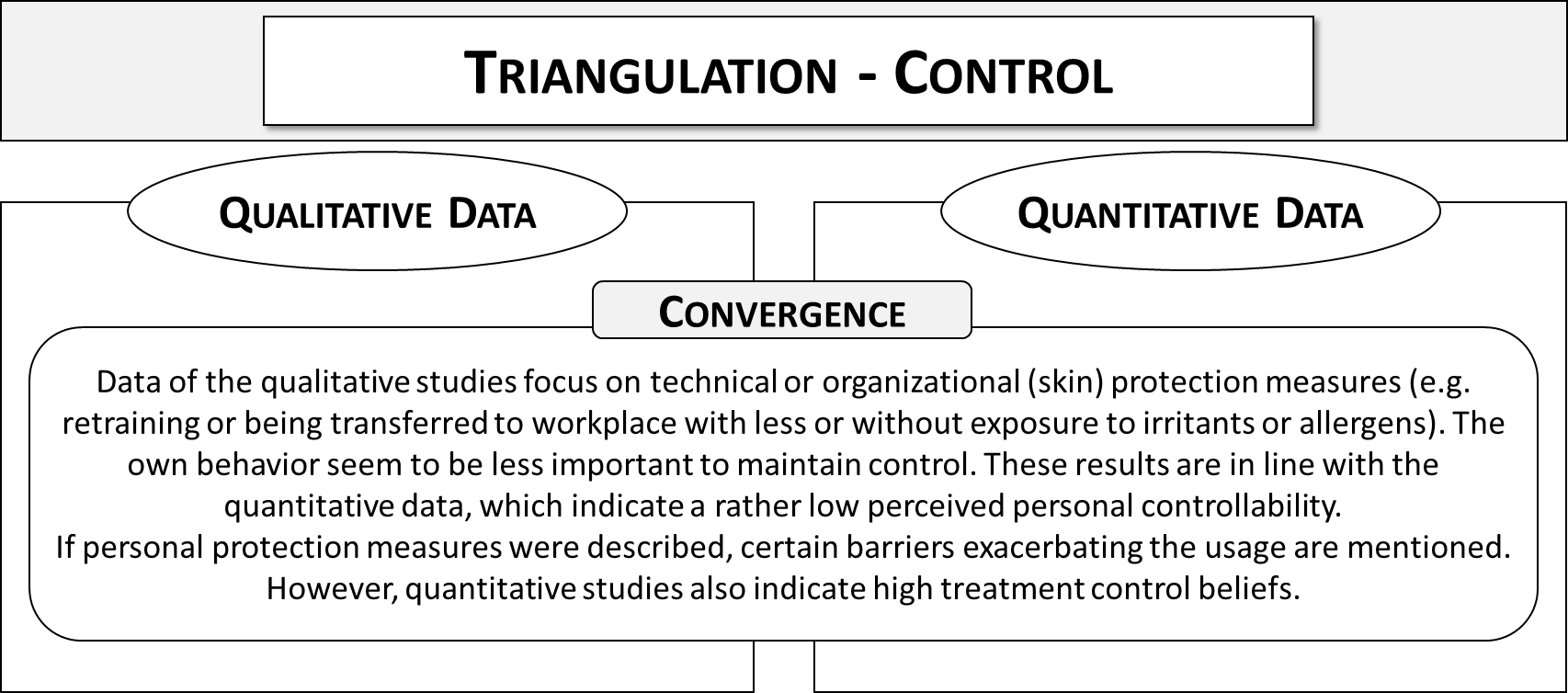


# Triangulation – Coherence


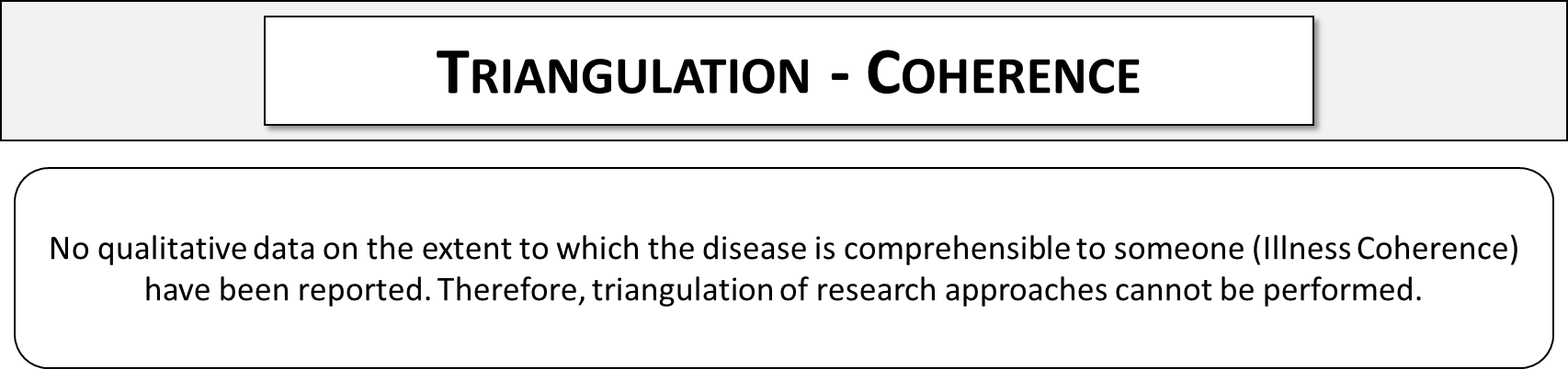


# Triangulation – Emotional Representation

*
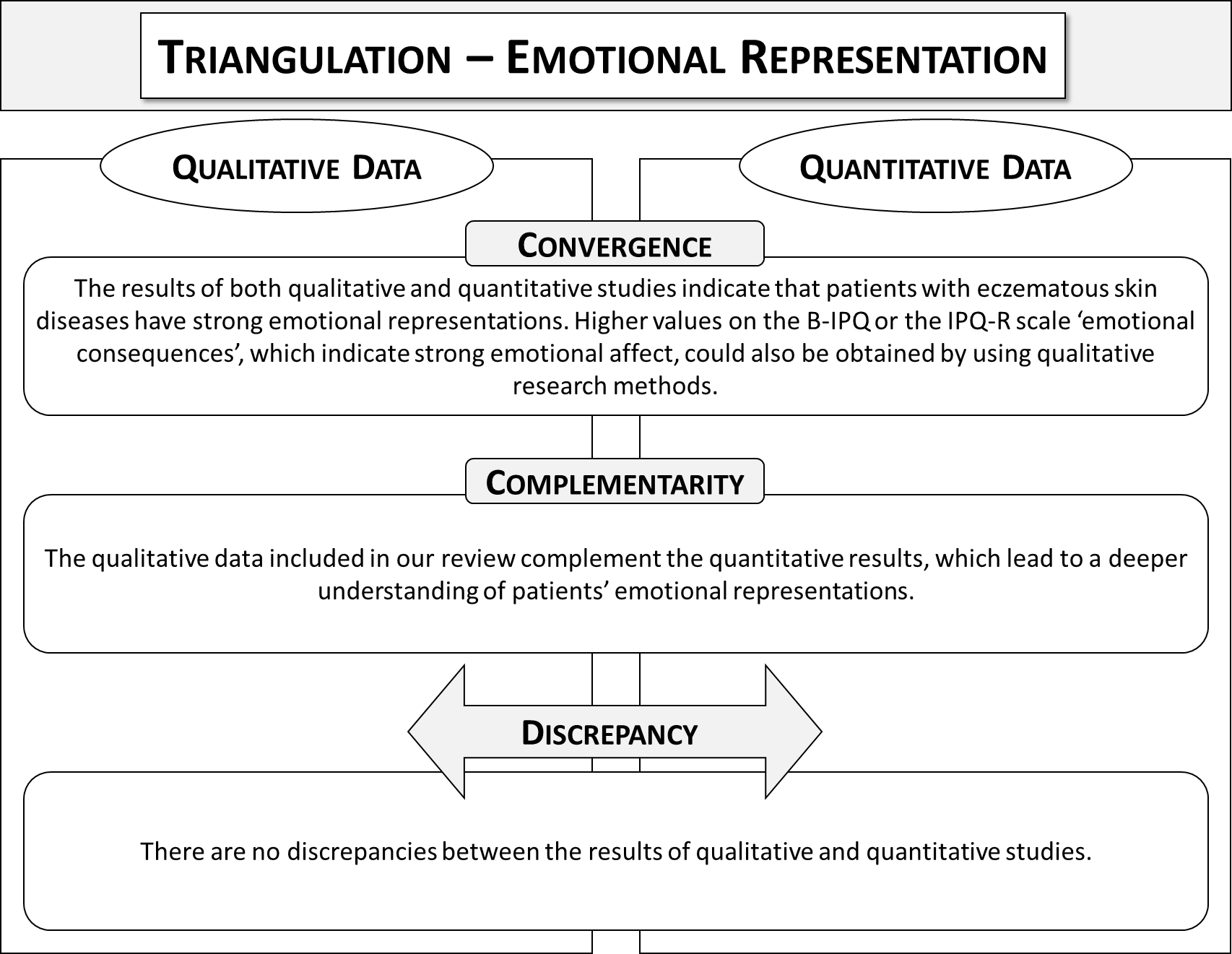
*
